# Solar exergy evaluation and empirical model establishment; case study: Iran

**DOI:** 10.1016/j.heliyon.2020.e05638

**Published:** 2020-12-04

**Authors:** Hossein Khorasanizadeh, Mojtaba Sepehrnia

**Affiliations:** a Faculty of Mechanical Engineering and the Energy Research Institute, University of Kashan, Kashan, Iran; b Department of Mechanical Engineering, Shahabdanesh University, Qom, Iran

**Keywords:** Energy, Renewable energy resources, Solar energy, Renewable energy, Solar exergy, Exergy to energy ratio, Sunshine duration, Empirical models, Iran

## Abstract

Iran with 300 sunny days in more than two thirds of its land is among the countries with high potential of solar energy. Nevertheless, to date no research has been conducted on status of solar exergy in Iran. In this study, in order to expand the perception of solar energy quality and to compensate the lack of research on solar radiation exergy in Iran, long term meteorological and solar data of eight capital provinces of Iran with five different climatic conditions are utilized. These properly distributed stations include Urmia, Bushehr, Isfahan, Ilam, Kerman, Mashhad, Zahedan and Zanjan. The monthly average daily solar radiation exergy on a horizontal surface for each station is obtained first, then it is recognized that the ratio of exergy to energy is almost independent of the month, the climatic condition and the geographical location; thus, can be considered 0.87 for the whole Iran. For predicting the solar exergy at every station, five empirical models with linear, quadratic, cubic, exponential and power functional forms, all dependent only on relative sunshine duration, are calibrated. Then, eight statistical indicators are utilized to evaluate the performance of the established models for every capital province. The best models recognized for Urmia, Bushehr, Isfahan, Ilam, Kerman, Mashhad, Zahedan and Zanjan have cubic, power, exponential, exponential, linear, quadratic, power and cubic functional forms, respectively. These models are simple and easy to apply and can be also utilized for other places with similar climatic classification and conditions.

## 1 Introduction

Solar energy is considered a clean source for energy supply to generate electricity and heat, because it does not produce any pollutants and does not endanger the environment. It has the highest energy level among all renewable sources. Solar energy data are fundamental and essential for researchers to design solar systems such as solar thermal systems, solar thermal-electric systems and photovoltaic systems. One of the most important information about solar energy is the monthly average daily solar radiation. So far, many studies have been carried out on the monthly average daily solar radiation measurement, prediction and model development or establishment. Some studies goes back to almost 100 years ago, by which efforts were made to know the important parameters affecting the solar radiation arriving on the earth (Angstrom, 1924).

The Angstrom-Prescott model (Prescott, 1940) has been used in most of the studies related to the monthly average daily solar radiation measurement and prediction (Almorox and Hontoria, 2004; Bakirci, 2009; Tasdemiroglu and Sever, 1991). In the Angstrom-Prescott model, the monthly average daily solar radiation is only dependent on the monthly average relative sunshine duration (*n*/*N*). There are some other meteorological parameters such as air temperature, relative humidity and water vapor and sea level pressures, which may affect the arriving monthly average solar radiation on the earth. In some studies, in addition to sunshine duration, either some of these parameters have been considered as extra variables (Akinoǧlu and Ecevit, 1990) or simple Angstrom-Prescott model has been modified (Khorasanizadeh and Mohammadi, 2013a, Khorasanizadeh and Mohammadi, 2013b). Also in some studies new methods like artificial neural networks has been utilized for global solar radiation estimation (Mohandes et al., 1998). However in a recent study (Mohammadi et al., 2016a), appraised the effects of adding different meteorological parameters including minimum, maximum and average air temperatures, maximum and average relative humidity, water vapor and sea level pressures to the simple Angstrom-Prescott model. It was shown that addition of various meteorological parameters to the Angstrom-Prescott model does not improve the accuracy of predictions significantly, but rather complicates it and therefore utilizing them is not suggested.

In general, unlike energy analysis it is the exergy analysis that discloses the degradation or destruction of energy from a useful form to an unwanted form during the conversion processes. Solar exergy determines the quality of the incoming solar energy and is the fraction of arriving solar energy that is convertible into electrical or mechanical energy. Electrical or mechanical energies are entirely exergy as they are completely convertible into all other energy types. Due to its entropy content, solar energy is not completely convertible; thus, its exergy content is less than 100% and is dependent on the atmospheric conditions (Kabelac, 2005). When a system does not hold any chemical potential and is in equilibrium with a reference environment at a particular temperature and pressure, it is at dead state and has zero exergy.

There are many studies reported in the literature, which are related to global or diffuse solar radiation predictions and the corresponding model development or establishment for different locations around the globe (Fan et al., 2020; Gouda et al., 2019; Khorasanizadeh and Mohammadi, 2016; Manju and Sandeep, 2019; Quej et al., 2017; Vakili et al., 2017), also for some Iranian stations (Khorasanizadeh and Mohammadi, 2013a, Khorasanizadeh and Mohammadi, 2013b; Khorasanizadeh and Mohammadi, 2013a, Khorasanizadeh and Mohammadi, 2013b; Khorasanizadeh et al., 2014a; Khorasanizadeh et al., 2014b; Mohammadi et al., 2016a); however studies performed on solar radiation exergy are scarce. Studies on solar exergy began on the basis of the second law of thermodynamics six decades ago and from then onwards the solar exergy has been an ongoing issue. Here, a passing reference is made only to some of the older studies with subjects of heat radiation exergy (Petela, 1964), conversion thermodynamics of solar radiation (Landsberg and Tonge, 1979), the efficiency of conversion (Bădescu, 1991; Parrott, 1978; Press, 1976) and application of exergy balance theory to solar collectors (Suzuki, 1988). However, a review of few solar exergy studies, which have been performed during the past twenty years, is presented in the following and references are made to their important achievements.

(Candau, 2003) studied the exergy of solar radiation and emphasized the importance of the second law of thermodynamics. It was shown that the analysis of exergy based on classical thermodynamics validates the results. In the same year (Koroneos et al., 2003), performed an exergy analysis of solar as well as wind power and geothermal energies, in which possible energy yields from these sources were discussed and the efficiency of utilization of each of these sources were compared with those of non-renewable sources.

Exergy analysis review and assessment of variety of renewable energy sources and systems, in particular thermal and photovoltaic solar energy systems were fulfilled by (Hepbasli, 2008). After performing a comprehensive review, it was stated that exergy analysis is a way to achieve sustainable development goals, because it can properly evaluate the performance of renewable energy systems.

(Alta et al., 2010) utilized the solar radiation data of 152 Turkish stations and mapped the spatial distribution of monthly mean solar radiation exergy over Turkey. They showed that the mean annual exergy-to-energy ratio for Turkey was 0.93 and the mean solar exergy per day was 13.5 ± 1.74 MJ/m^2^.

(Hepbasli and Alsuhaibani, 2014) conducted a study on the estimation and comparison of solar exergy in different climate areas of Turkey and Saudi Arabia. Two major goals were: 1-Comprehensive study of various models of exergy analysis to use in solar systems 2-Determination of solar exergy values for some regions of Turkey and Saudi Arabia. The mean annual exergy-to-energy ratio for northeastern Saudi Arabia and Izmir in Turkey were obtained as 0.933 and 0.935 according to Petela's approach, respectively. Also according to Jefer's approach, the mean annual exergy-to-energy ratio for northeastern Saudi Arabia and Izmir in Turkey were attained 0.950 and 0.951, respectively.

For predicting the monthly average solar exergy for seven stations in Turkey (Arslanoglu, 2016), established three linear, quadratic and cubic sunshine duration based models for every station. Also to evaluate the performance of the models, seven statistical indicators were utilized. However, Arslanoglu did not introduce a single model as the best model for each station and stated that all of the calibrated models for each station provided reliable results for the monthly average daily solar radiation exergy prediction in that station.

For estimating solar exergy in India (Jamil and Bellos, 2019), established models based on averaged diffuse and global solar radiation, air temperature and sunshine hours from 23 climate stations for a period of 25 years. Results showed that the power model based on clearness index was the best model to predict the global exergy efficiency factor.

Iran with 300 sunny days in more than two thirds of its land is among the countries with high potential of solar energy. Nevertheless, to date no research has been conducted on solar exergy status in Iran. In order to expand information about the general status of solar exergy in Iran and distribution of the ratio of solar exergy to solar energy around the globe, the main objective of this research is investigation of solar exergy potential at eight well distributed capital provinces of Iran. These stations are Urmia, Bushehr, Isfahan, Ilam, Kerman, Mashhad, Zahedan and Zanjan, which are the capitals of West Azerbaijan, Bushehr, Isfahan, Ilam, Kerman, Khorasan Razavi, Sistan & Baluchestan and Zanjan provinces, respectively. As stated in Table 1, based upon Koppen classification, these stations have five different climatic conditions of BWk, BSh, BWh, BSk and Csa. The long term meteorological and solar data of these stations are utilized to appraise their solar exergy status based on the second law of thermodynamics, first. Then as the second objective, five models with linear, quadratic, cubic, exponential and power functional forms all dependent only to relative sunshine duration are established for the monthly average daily solar exergy prediction. Afterward and as the third objective, the performances of these models are evaluated and the best model for each province is determined. In this study, different from the study of (Arslanoglu, 2016), eight statistical indicators are used and for each individual station the best model for predicting solar exergy is recognized and introduced. Similar to the Angstrom-Prescott model, which is to predict the total solar radiation, the best models of this study are dependent solely to the monthly average relative sunshine duration and independent of any other solar or metrological parameter; hence are simple and easy to apply and may be used for other regions around the globe, if they have similar climatic classification and conditions.

**Table 1:** Specification of the eight selected capital provinces.

Location	Province	Climatic classification	Latitude (North)	Longitude (East)	Elevation (m)	The period of data series
Urmia	West Azerbaijan	(BSk)	37°33ʹ	45°04ʹ	1348	1992–2016
Bushehr	Bushehr	(BSh)	28°57ʹ	50°50ʹ	11	2006–2016
Isfahan	Isfahan	(BWh)	32°40ʹ	51°40ʹ	1575	1992–2016
Ilam	Ilam	(Csa)	33°38ʹ	46°24ʹ	1369	2003–2016
Kerman	Kerman	(BWk)	30°17ʹ	57°04ʹ	1764	1992–2016
Mashhad	Khorasan Razavi	(BSk)	36°19ʹ	59°32ʹ	1027	1992–2016
Zahedan	Sistan & Baluchestan	(BWh)	29°30ʹ	60°51ʹ	1386	1992–2016
Zanjan	Zanjan	(BSk)	36°40ʹ	48°29ʹ	1678	1992–2016

## 2 Material and methods

In this section, the content is presented through four subsections of the study region and data collection, solar modeling, solar exergy predicting models and statistical indicators, respectively.

### 2.1 The study region and data collection

**Figure 1:**
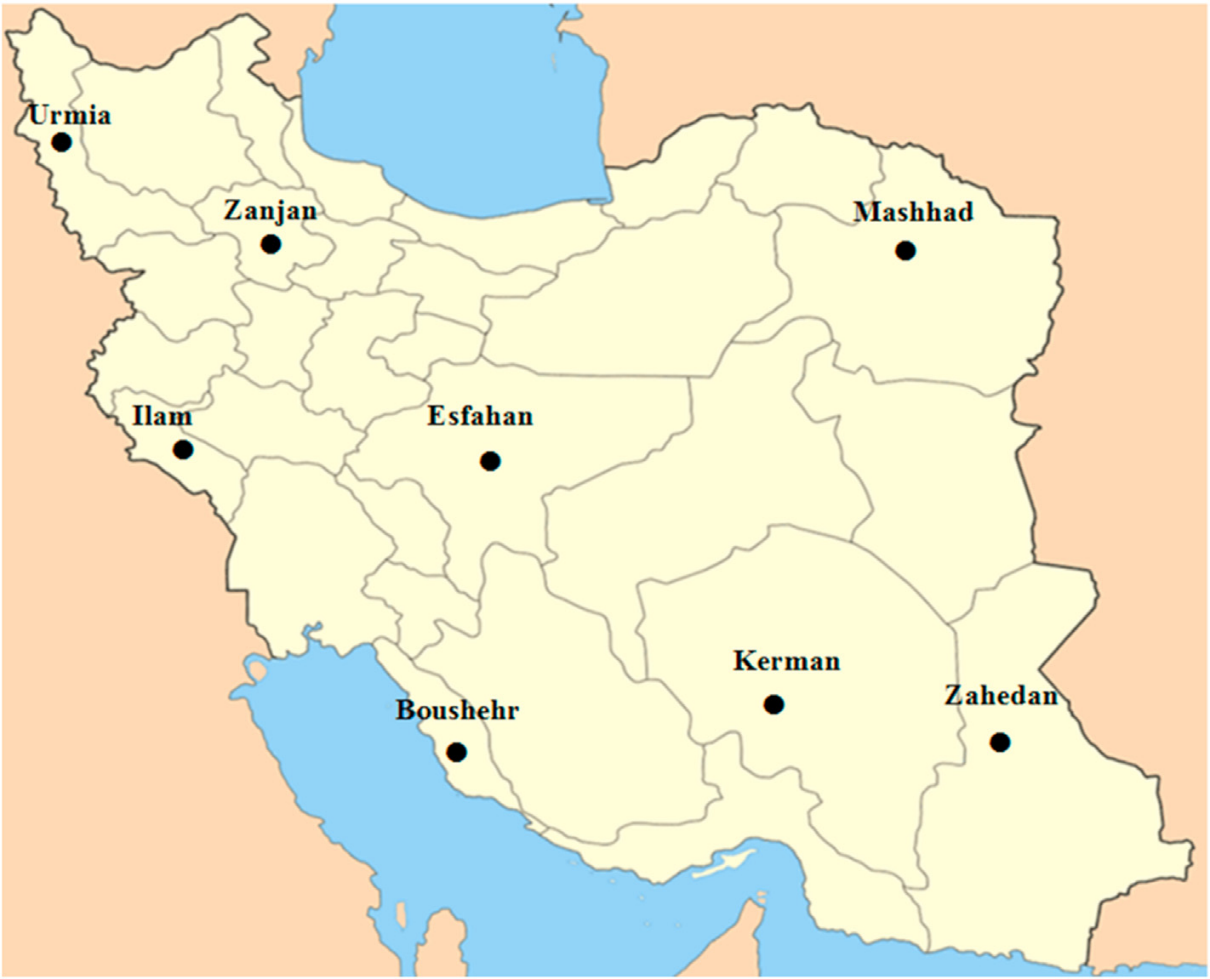
Location of the studied capital provinces on the Iranian map.

Iran is located between 25°03′ and 39°47′ north latitude and 44°05′ and 63°18′ eastern longitude and is a relatively high country, with an average elevation of over 1000 m above the sea level. In this study, eight province capitals have been selected with sufficient distribution, such that involve latitudes between 28°57′ and 37°33′ and longitudes between 45°04′ and 60°51'. The location of the studied stations on the map of the Iran has been shown in Figure 1. In Table 1, the information about the eight selected stations including the provinces names, climatic classification, longitude, latitude, elevation from the sea level and the meteorological station period of data series, provided by the Iranian Meteorological Organization (IMO), have been presented.

Meteorological station data series involved the daily ambient temperature, daily sunshine duration and daily solar radiation. In order to enhance the quality of global solar radiation data, two important points were considered:

1. In order to refine the global solar radiation values, all values resulting a daily clearness index out of range of (0.015 < *K*_T_ < 1) were eliminated.
2. Global solar radiation data collections for months which involved more than five days incorrect, missing or unavailable data were eliminated.

After performing data refinement, the long term data of each selected capital station were used to obtain the monthly average daily relative sunshine duration, monthly average daily ambient temperature and monthly average daily solar radiation of that station for 12 months of the year. These monthly average daily values have been presented for the eight selected locations in Table 2 and the maximum value of each item for every month has been shown in bold. The results show that Isfahan in February, March, May, June and September, Zahedan in January, April and November, Kerman in October and December and Mashhad in July and August have the highest monthly average relative sunshine duration values compared with those of other stations at the same months, indicating that these stations enjoy from more sunny hours and sunny days at these months. However, the highest monthly average daily ambient temperature values throughout the year belong to Bushehr. Kerman in 9 months of the year from March to November and Zahedan in 3 months of January, February and December have the maximum monthly average daily solar radiation values compared with those of other stations at the same months.

**Table 2:** Monthly average daily relative sunshine duration, monthly average daily ambient temperature (K) and monthly average daily solar radiation (kJ/m^2^) in the eight selected locations of Iran.

Location	Parameter	January	February	March	April	May	June	July	August	September	October	November	December
Urmia	n/N	0.476	0.568	0.568	0.585	0.653	0.799	0.829	0.847	0.801	0.677	0.605	0.461
	T_o_	271.71	274.15	278.99	284.59	289.24	294.09	297.09	296.71	292.15	285.86	278.82	273.60
	H	6433.35	6929.61	9856.02	10770.56	14028.66	15116.19	15577.83	12443.46	11321.89	8649.96	6498.45	6249.97
Boushehr	n/N	0.709	0.708	0.622	0.637	0.693	0.791	0.727	0.768	0.795	0.796	0.712	0.712
	T_o_	**289.65**	**290.78**	**294.38**	**297.80**	**303.11**	**305.15**	**307.05**	**307.95**	**305.98**	**302.12**	**295.48**	**291.32**
	H	9809.40	9258.30	12853.01	10466.76	15143.26	16772.3	15089.97	14429.95	12432.57	11118.52	9422.31	8978.69
Isfahan	n/N	0.647	**0.732**	**0.702**	0.678	**0.755**	**0.835**	0.821	0.873	**0.877**	0.829	0.712	0.639
	T_o_	276.52	280.09	284.68	290.17	295.57	300.80	303.37	301.43	297.36	291.01	283.05	278.08
	H	8238.64	9504.71	11943.63	13302.90	17153.33	16910.08	17573.14	16340.95	14221.67	10955.77	8491.49	8169.60
Ilam	n/N	0.540	0.602	0.618	0.616	0.635	0.806	0.798	0.832	0.857	0.720	0.661	0.587
	T_o_	277.20	278.95	283.45	288.06	293.90	299.86	302.56	302.32	297.61	291.81	283.41	279.22
	H	8484.12	9052.15	14033.46	15598.11	17957.46	18879.71	19324.82	18180.3	14843.97	10898.93	8896.87	7454.72
Kerman	n/N	0.674	0.678	0.658	0.679	0.733	0.794	0.803	0.870	0.864	**0.859**	0.785	**0.736**
	T_o_	278.07	280.97	285.35	290.79	296.26	300.02	301.78	299.46	296.10	290.55	283.80	279.48
	H	10221.49	11026.89	**14073.72**	**16074.68**	**19811.47**	**20157.93**	**21222.8**	**19969.92**	**17503.99**	**14315.32**	**11084.89**	9738.80
Mashhad	n/N	0.498	0.506	0.483	0.560	0.659	0.778	**0.855**	**0.884**	0.841	0.746	0.598	0.512
	T_o_	275.81	277.89	282.75	288.85	294.61	299.49	301.47	299.92	295.02	288.69	281.95	277.52
	H	7261.81	8546.36	11595.15	14162.98	16281.89	17607.47	19313.63	17017.48	15026.73	11382.04	7711.89	6940.72
Zahedan	n/N	**0.725**	0.727	0.663	**0.707**	0.723	0.775	0.813	0.852	0.860	0.856	**0.837**	0.722
	T_o_	280.56	283.69	288.58	294.31	299.21	302.29	303.35	301.40	297.35	292.15	286.29	281.68
	H	**11504.7**	**12032.94**	12599.52	15180.05	17931.33	16845.61	18816.61	18059.57	15369.28	13162.68	10567.41	**9900.04**
Zanjan	n/N	0.491	0.547	0.547	0.566	0.650	0.776	0.798	0.835	0.835	0.716	0.569	0.484
	T_o_	271.42	273.71	278.48	284.20	288.76	293.75	296.91	296.77	291.95	285.77	278.68	274.20
	H	6344.71	7344.42	10458.65	12101.08	14461.65	16863.33	16334.25	14681.19	12589.63	9439.07	6235.80	6534.73

### 2.2 Solar modeling

H_o_ is the daily extraterrestrial solar radiation on a horizontal surface expressed as (Duffie and Beckman, 2013):

(1) $$H_{O}=\frac{24\text{×}3600\text{×}{\text{G}}_{\text{SC}}}{\pi }\left[1+0.033\;\cos\left(\frac{360D}{365}\right)\right]\left[\cos\;\varphi \;\cos\;\delta \;\sin\;\omega_{s}+\frac{2\text{π}{\text{ω}}_{\text{s}}}{360}\sin\;\varphi \;\sin\;\delta \right]$$

where G_sc_ is the solar constant equal to 1367 W/m^2^ (Duffie and Beckman, 2013). *φ* is the latitude and δ is the solar declination angle. The solar declination angle changes between -23.45 on December 2 to 23.45 on June 21 (Duffie and Beckman, 2013). *ω*_s_ and *D* are the sunset hour angle and number of day counted from first of January, respectively.

The declination angle is obtained via (Duffie and Beckman, 2013):

(2) $$\delta =23.45\;\sin\left[\frac{360\left(D+284\right)}{365}\right]$$

Sunset hour angle for a horizontal surface is expressed as (Duffie and Beckman, 2013):

(3) $$\omega_{s}=\cos^{-1}\left[-\tan\;\delta \;\tan\;\varphi \right]$$

The monthly average day length is calculated by (Duffie and Beckman, 2013):

(4) $$N=\frac{2\omega_{s}}{15}$$

The actual efficiency of a solar system is the ratio of the work performed by the system divided by the solar radiation energy as (Alta et al., 2010; Hepbasli and Alsuhaibani, 2014):

(5) $$\eta_{e}=\frac{W}{E_{rad}}$$

In a reversible process, the maximum work is obtained from the solar radiation energy. According to Petela (2003), such work is equivalent to solar radiation exergy. Therefore, the maximum efficiency is (Alta et al., 2010; Hepbasli and Alsuhaibani, 2014):

(6) $$\eta_{e,\max}=\frac{Ex_{rad}}{E_{rad}}=\psi$$

$Ex_{rad}$ and ψ in Eq. (6) are the radiation exergy and the ratio of exergy to energy, respectively. The ratio of exergy to energy for solar radiation is calculated via the following equation (Petela, 2005):

(7) $$\psi \left(T_{o}\right)=1+\frac{1}{3}{\left(\frac{T_{o}}{T_{s}}\right)}^{4}-\frac{4}{3}\frac{T_{o}}{T_{s}}$$

In Eq. (7), *T*o is the monthly average daily ambient temperature and *T*s is the sun temperature (6000K), if the sun is assumed to be a black body (Hepbasli, 2008).

By replacing the monthly average daily solar radiation, *H*, as solar radiation energy, instead of *E*_*rad*_ in Eq. (6) and then rearrangement, the monthly average daily solar exergy, *H*_*Exergy*_, becomes:

(8) $$H_{Exergy}=Ex_{rad}=\psi \left(T_{o}\right)H$$

Regarding the relation between equation (7) and equation (8), the monthly average daily solar exergy depends on the monthly average daily ambient temperature and monthly average daily solar radiation. If Eq. (8) is divided by the monthly average daily extraterrestrial radiation on a horizontal surface it results in:

(9) $$\frac{H_{Exergy}}{H_{O}}=\psi \left(T_{o}\right)\frac{H}{H_{O}}=\psi \left(T_{o}\right)f\left(a,b,c,d,n,N\right)$$

However, if regression models, which are based only on monthly average relative sunshine duration, are developed to predict the monthly average daily solar radiation exergy normalized by the monthly average daily extraterrestrial radiation, these models become independent of the monthly average daily ambient temperature and monthly average daily solar radiation, such that:

(10) $$\frac{H_{Exergy}}{H_{O}}=f\left(a^{\prime },b^{\prime },c^{\prime },d^{\prime },n,N\right)$$

The functional forms of solar exergy models considered in this study are presented in the next subsection and the details of model development for the eight selected stations of Iran is explained in section 3.2.

### 2.3 Solar exergy predicting models

So far, for global or diffuse solar radiation predictions many empirical correlations with different functional forms have been developed or established, in which different solar or metrological parameters have been utilized as variables. In 2016 (Mohammadi et al., 2016a), showed that addition of various meteorological parameters to the Angstrom-Prescott model, which in its conventional form is a linear function of relative sunshine duration, does not improve the accuracy of the global solar radiation prediction. Also, in another study, aimed at recognizing the most relevant variables for diffuse solar radiation prediction (Mohammadi et al., 2016b), recognized that sunshine duration (*n*) is the most influential variable. Thus, in order to establish empirical models for predicting the monthly average daily solar radiation exergy in the eight selected stations of Iran, in this study five linear, quadratic, cubic, exponential and power relations, all dependent only to the monthly average relative sunshine duration, were considered as:

(11) $$\frac{H_{Exergy}}{H_{O}}=a^{\prime }+b^{\prime }\left(n/N\right)$$

(12) $$\frac{H_{Exergy}}{H_{O}}=a^{\prime }+b^{\prime }\left(n/N\right)+c^{\prime }{\left(n/N\right)}^{2}$$

(13) $$\frac{H_{Exergy}}{H_{O}}=a^{\prime }+b^{\prime }\left(n/N\right)+c^{\prime }{\left(n/N\right)}^{2}+d^{\prime }{\left(n/N\right)}^{3}$$

(14) $$\frac{H_{Exergy}}{H_{O}}=a^{\prime }e^{b^{\prime }\left(n/N\right)}$$

(15) $$\frac{H_{Exergy}}{H_{O}}=a^{\prime }{\left(n/N\right)}^{b^{\prime }}$$

### 2.4 Statistical indicators

To evaluate the performance of the calibrated models eight statistical indicators of mean bias error (*MBE*), mean absolute bias error (*MABE*), mean percentage error (*MPE*), mean absolute percentage error (*MAPE*), root mean square error (*RMSE*), relative root mean square error (*RRMSE*), t-statistics (*t*_sta_) and correlation coefficient (*R*^2^) have been utilized.

*MBE* shows the long-term performance of the models. The ideal value for *MBE* is zero. This indicator is expressed as (Khorasanizadeh et al., 2014a):

(16) $$MBE=\frac{1}{k}\sum_{i=1}^{k}H_{\mathrm{Pr}ed,Exergy}-H_{Exergy}$$

*MABE* determines the absolute value of the bias error. The ideal value for *MABE* is zero. This indicator is expressed as (Khorasanizadeh et al., 2014b):

(17) $$MABE=\frac{1}{k}\sum_{i=1}^{k}\left|H_{\mathrm{Pr}ed,Exergy}-H_{Exergy}\right|$$

*MPE* defines the average relative error percentage. The ideal value for *MPE* is zero. This indicator is defined as (Teke and Yıldırım, 2014):

(18) $$MPE=\frac{1}{k}\sum_{i=1}^{k}\left(\frac{H_{\mathrm{Pr}ed,Exergy}-H_{Exergy}}{H_{Exergy}}\times 100\right)$$

*MAPE* determines the absolute value of the average relative error percentage. The ideal value for *MAPE* is zero. This indicator is defined as follows (Khorasanizadeh and Mohammadi, 2013a, Khorasanizadeh and Mohammadi, 2013b):

(19) $$MAPE=\frac{1}{k}\sum_{i=1}^{k}\left(\frac{H_{\mathrm{Pr}ed,Exergy}-H_{Exergy}}{H_{Exergy}}\times 100\right)$$

*RMSE* gives good information about the short-term performance of models. The value of this indicator is always greater than or equal to zero. The ideal value for *RMSE* is zero. This indicator is expressed (Khorasanizadeh and Mohammadi, 2013a, Khorasanizadeh and Mohammadi, 2013b):

(20) $$RMSE={\left[{\frac{1}{k}\sum_{i=1}^{k}(H_{\mathrm{Pr}ed,Exergy}-H_{Exergy})}^{2}\right]}^{0.5}$$

*RRMSE* is obtained by dividing the *RMSE* by the average calculated exergy. This indicator is calculated via (Khorasanizadeh et al., 2014a):

(21) $$RRMSE=\frac{{\left[{\frac{1}{k}\sum_{i=1}^{k}(H_{\mathrm{Pr}ed,Exergy}-H_{Exergy})}^{2}\right]}^{0.5}}{\frac{1}{k}\sum_{i=1}^{k}H_{Exergy}}\times 100$$

*RRMSE* clarifies the accuracy of the models according to the following classification (Jamieson et al., 1991; Li et al., 2013):

Very good accuracy: *RRMSE* < 10%.

Good accuracy: 10% < *RRMSE* < 20%.

Medium accuracy: 20% < *RRMSE* < 30%.

Poor accuracy: *RRMSE* > 30%.

*t*_sta_ contains two indicators of *MBE* and *RMSE* and is expressed as:

(22) $$t_{sta}=\sqrt{\frac{\left(n-1\right)MBE^{2}}{RMSE^{2}-MBE^{2}}}$$

The smaller *t*_sta_ value shows better performance of a model. *n*-1 is the degrees of freedom and in this study *n*-1 = 11.

*R*^2^ expresses the linearity of the relationship between the calculated and predicted values and varies between -1 and +1. The values of ±1 show the complete linearity of the relationship between the calculated and predicted values and the value of 0 indicates the absence of a linear relationship. This indicator is defined as follows (Khorasanizadeh et al., 2014b):

(23) $$R^{2}=\frac{\sum_{i=1}^{k}\left(H_{\mathrm{Pr}ed,Exergy}-H_{\mathrm{Pr}ed,Exergy,Avg}\right)\left(H_{Exergy}-H_{Exergy,Avg}\right)}{\sqrt{\left[\sum_{i=1}^{k}{\left(H_{\mathrm{Pr}ed,Exergy}-H_{\mathrm{Pr}ed,Exergy,Avg}\right)}^{2}\right]\left[\sum_{i=1}^{k}{\left(H_{Exergy}-H_{Exergy,Avg}\right)}^{2}\right]}}$$

## 3 Results and discussion

In this section the status of solar exergy in the eight selected capital provinces of Iran is reviewed and discussed first. Then the results related to establishment of five different models for predicting the solar exergy in these stations are presented. At last based on the statistical indicators the best model for each station is recognized and introduced.

### 3.1 Status of solar radiation exergy in Iran

**Figure 2:**
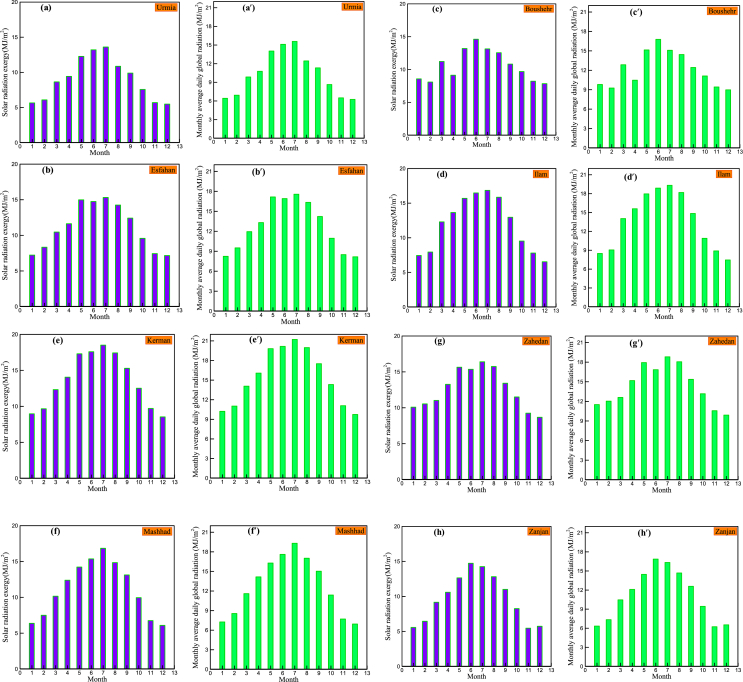
The monthly average daily solar exergy in: a) Urmia, b) Isfahan, c) Bushehr, d) Ilam, e) Kerman, f) Mashhad, g) Zahedan and h) Zanjan and the monthly average daily solar radiation in: aʹ) Urmia, bʹ) Isfahan, cʹ) Bushehr, dʹ) Ilam, eʹ) Kerman, fʹ) Mashhad, gʹ) Zahedan and hʹ) Zanjan.

Based on the long term measured data, for all of the months of the year the monthly average daily solar radiation exergy have been calculated via Eqs. (7) and (8). Figure 2 shows the monthly average daily solar exergy and the monthly average daily solar radiation in the eight selected stations of Iran. The results show that in all of the stations the maximum monthly average daily solar exergy and the maximum monthly average daily solar radiation occur in June or July, and the minimum monthly average daily solar exergy and minimum monthly average daily solar radiation occur either in November or in December. In the three stations of Ilam, Kerman and Mashhad, variation of the monthly average daily solar radiation exergy is harmonic; it increases gradually from January, reaches to its peak in July and then declines toward December. The maximum monthly average daily solar exergy and the maximum monthly average daily solar radiation are 18.51 MJ/m^2^day and 21.22 MJ/m^2^day respectively in Kerman on July and the minimums are 5.47 MJ/m^2^day and 6.23 MJ/m^2^day respectively in Zanjan on November.

In Table 3 the monthly average daily ratio of solar exergy to solar energy for the eight selected stations have been presented. The results show that the average ratio of exergy to energy is almost independent of the month and geographical location; therefore, the average ratio of exergy to energy for the whole Iran can be considered 0.87. It should be noted that in the study of (Alta et al., 2010) and (Arslanoglu, 2016) both performed for Turkey, this ratio was found to be 0.93. Also in study of (Hepbasli and Alsuhaibani, 2014) the mean annual exergy to energy ratio for northeastern Saudi Arabia and Izmir in Turkey were obtained 0.933 and 0.935 according to Petela's approach, respectively and 0.950 and 0.951 according to Jefer's approach, respectively. The discrepancies are due to differences associated with the atmospheric and climatic conditions of the mentioned stations compared with those of Iran.

**Table 3:** The monthly average ratio of solar exergy to solar energy in the eight selected locations of Iran.

Location	January	February	March	April	May	June	July	August	September	October	November	December
Urmia	0.879	0.878	0.877	0.876	0.875	0.874	0.873	0.873	0.874	0.876	0.877	0.879
Boushehr	0.875	0.875	0.874	0.873	0.872	0.872	0.871	0.871	0.871	0.872	0.874	0.875
Isfahan	0.878	0.877	0.876	0.875	0.874	0.872	0.872	0.872	0.873	0.8745	0.876	0.878
Ilam	0.878	0.877	0.876	0.875	0.874	0.873	0.872	0.872	0.873	0.874	0.876	0.877
Kerman	0.878	0.877	0.876	0.875	0.873	0.873	0.872	0.873	0.873	0.875	0.876	0.877
Mashhad	0.878	0.878	0.876	0.875	0.874	0.873	0.872	0.873	0.874	0.875	0.877	0.878
Zahedan	0.877	0.876	0.875	0.874	0.873	0.872	0.872	0.872	0.873	0.874	0.876	0.877
Zanjan	0.879	0.878	0.877	0.876	0.875	0.874	0.873	0.873	0.874	0.876	0.877	0.878

### 3.2 Development of solar exergy models

The monthly average daily extraterrestrial solar radiation for the eight selected stations, obtained via Eq. (1), have been presented in Table 4. Among all of the locations, the maximum solar radiation in each month has been shown in bold. Bushehr in nine months of January, February, March, April, August, September, October, November and December, Zahedan in February, Isfahan in May, Urmia in June and Mashhad in July have the maximum monthly average daily extraterrestrial radiation among all stations. Generally, monthly average daily extraterrestrial radiation for the three stations of Bushehr, Kerman and Zahedan are higher than those of other stations.

**Table 4:** The monthly average daily extraterrestrial radiation in the eight selected locations of Iran (kJ/m^2^).

Location	January	February	March	April	May	June	July	August	September	October	November	December
Urmia	15941.5	20925.7	28412.2	34366.1	41265.1	**41733.2**	43252.0	38166.7	30160.1	23835.9	18018.7	15304.8
Boushehr	**20865.2**	**25291.8**	**31818.9**	**36043.1**	41390.0	41054.6	42914.4	**39246.8**	**32861.8**	**28042.8**	**23055.3**	**20573.0**
Isfahan	18776.7	23471.3	30438.5	35415.4	**41430.3**	41430.4	43152.0	38879.7	31788.1	26302.5	20928.0	18328.1
Ilam	18358.2	23100.9	30150.3	35274.5	41421.3	41489.5	43182.2	38789.6	31560.0	25945.9	20500.1	17880.1
Kerman	20123.7	24650.8	31339.9	35834.9	41420.9	41204.0	43015.7	39132.5	32493.1	27432.4	22301.5	19774.3
Mashhad	16239.0	21196.9	28633.0	34486.6	41293.7	41711.8	**43252.7**	38252.7	30340.0	24100.4	18325.2	15620.8
Zahedan	20568.3	25035.8	31628.5	35961.7	41404.6	41116.6	42957.3	39203.2	32715.8	27799.4	22753.7	20253.0
Zanjan	16454.6	21392.7	28791.8	34572.3	41312.7	41694.8	43251.7	38313.4	30469.1	24291.2	18547.1	15849.9

After calculating the ratio of *H*_*Exergy*_ to *H*_o_ for all of the months of the year for all of the selected stations, the monthly average daily sunshine duration data were used to establish five new models for predicting the monthly average daily solar radiation exergy via utilizing regression analysis. The regression constants of the five calibrated linear, quadratic, cubic, exponential and power models for the eight selected stations have been presented in Table 5.

**Table 5:** The regression constants of the calibrated models for predicting the monthly average daily solar radiation exergy in the eight selected stations of Iran.

Location	Models	$a^{\prime }$	$b^{\prime }$	$c^{\prime }$	$d^{\prime }$
Urmia	Linear	0.357	-0.067		
	Quadratic	0.769	-1.357	0.972	
	Cubic	4.024	-17.283	26.337	-13.146
	Exponential	0.355	-0.196		
	Power	0.293	-0.146		
Boushehr	Linear	0.236	0.141		
	Quadratic	-1.208	4.198	-2.833	
	Cubic	14.741	-63.703	93.112	-44.996
	Exponential	0.232	0.513		
	Power	0.380	0.380		
Isfahan	Linear	0.351	0.016		
	Quadratic	1.751	-3.710	2.450	
	Cubic	6.807	-24.031	29.523	-11.948
	Exponential	0.348	0.053		
	Power	0.365	0.027		
Ilam	Linear	0.332	0.080		
	Quadratic	0.921	-1.624	1.205	
	Cubic	1.372	-3.624	4.129	-1.408
	Exponential	0.334	0.210		
	Power	0.407	0.137		
Kerman	Linear	0.233	0.257		
	Quadratic	0.284	0.122	0.088	
	Cubic	-4.117	17.521	-22.667	9.882
	Exponential	0.270	0.605		
	Power	0.487	0.461		
Mashhad	Linear	0.323	0.087		
	Quadratic	0.479	-0.397	0.357	
	Cubic	1.740	-6.209	9.077	-4.266
	Exponential	0.327	0.224		
	Power	0.404	0.143		
Zahedan	Linear	0.344	0.075		
	Quadratic	-1.652	5.275	-3.362	
	Cubic	-41.676	164.941	-214.82	92.947
	Exponential	0.335	0.233		
	Power	0.421	0.190		
Zanjan	Linear	0.288	0.063		
	Quadratic	0.839	-1.659	1.2939	
	Cubic	3.791	-15.643	22.973	-10.998
	Exponential	0.289	0.194		
	Power	0.345	0.111		

### 3.3 Introducing the best model for every station

To evaluate the performance of the calibrated models for the eight selected locations the statistical indicators, introduced in section 2.4, have been attained and the results have been presented in Table 6. The best model according to each individual statistical indicator has been shown in bold in Table 6. However, as explained in the following subsections, after observing all of the statistical indicators, the best model for each station has been recognized. The best models presented in Table 7, can be utilized for the selected stations as well as for other regions around the globe with similar climatic classification and conditions as those of the nominated stations. It should be noted that the climatic classification and conditions of the selected stations have been presented in Table 1 and Table 2.

**Table 6:** The statistical indicators of the five calibrated exergy models for the eight selected locations of Iran.

Location	Indicators	Models				
		Linear	Quadratic	Cubic	Exponential	Power
Urmia	*MBE*	0.0667	0.0519	**0.0343**	0.0530	0.0398
	*MABE*	0.5591	0.4483	**0.2648**	0.5543	0.5546
	*MPE*	0.5148	0.3710	**0.0710**	0.3121	0.2590
	*MAPE*	6.3522	5.1004	**2.9580**	6.3363	6.2815
	*RMSE*	0.6568	0.5861	**0.3134**	0.6466	0.6455
	*RRMSE*	7.2672	6.4848	**3.4683**	7.1547	7.1426
	*t*_*sta*_	0.3385	0.29501	0.3657	0.2731	**0.2049**
	*R*^*2*^	0.9752	0.9804	**0.9948**	0.9750	0.9756
Boushehr	*MBE*	0.1931	0.1729	0.1790	0.1299	**0.1289**
	*MABE*	0.9052	0.9629	0.9633	**0.8939**	0.8963
	*MPE*	1.2810	1.1972	1.1840	0.6526	**0.6409**
	*MAPE*	9.0766	9.3269	9.2904	**9.0018**	9.0120
	*RMSE*	1.1191	1.1407	1.1420	1.0951	**1.0939**
	*RRMSE*	10.5572	10.7609	10.7740	10.3312	**10.3194**
	*t*_*sta*_	0.5809	0.5086	0.5264	0.3962	**0.3935**
	*R*^*2*^	0.9195	0.9127	0.9158	0.9219	**0.9222**
Isfahan	*MBE*	0.4339	**0.2607**	0.3113	0.4167	0.4237
	*MABE*	0.5170	0.6931	0.7128	**0.5087**	0.5130
	*MPE*	4.7276	**3.2282**	3.5814	4.5691	4.6378
	*MAPE*	5.3924	6.3618	6.4770	**5.2964**	5.3555
	*RMSE*	0.6613	0.9506	1.0389	**0.6483**	0.6576
	*RRMSE*	5.9401	8.5385	9.3317	**5.8229**	5.9064
	*t*_*sta*_	2.8839	**0.9459**	1.0416	2.7836	2.7943
	*R*^*2*^	0.9878	0.9546	0.9461	**0.9880**	0.9876
Ilam	*MBE*	0.0217	-0.2555	-0.2312	**0.0084**	0.0137
	*MABE*	**0.4273**	0.5113	0.4985	0.4309	0.4318
	*MPE*	0.9631	-1.4922	-1.2899	**0.8484**	0.9015
	*MAPE*	**4.2693**	4.5686	4.5063	4.2811	4.2916
	*RMSE*	**0.5219**	0.5790	0.5691	0.5227	0.5235
	*RRMSE*	**4.378**	4.8568	4.7742	4.3845	4.3918
	*t*_*sta*_	0.1381	1.6309	1.4745	**0.0530**	0.0865
	*R*^*2*^	0.9911	**0.9921**	0.9919	0.9911	0.9910
Kerman	*MBE*	**-0.2580**	-0.2594	-0.3061	-0.2702	-0.2674
	*MABE*	**0.4466**	0.4562	0.4656	0.4564	0.4474
	*MPE*	**-1.9971**	-2.0032	-2.3141	-2.0862	-2.0735
	*MAPE*	**3.5486**	3.6205	3.7221	3.6210	3.5600
	*RMSE*	0.5145	0.5211	0.5341	0.5241	**0.5125**
	*RRMSE*	3.8128	3.8616	3.9576	3.8838	**3.7978**
	*t*_*sta*_	1.9218	**1.9035**	2.3194	1.9952	2.0285
	*R*^*2*^	0.9922	0.9919	0.9924	0.9920	**0.9924**
Mashhad	*MBE*	0.2114	**0.0412**	0.1474	0.1847	0.2085
	*MABE*	0.5750	0.5434	**0.4917**	0.5687	0.5791
	*MPE*	1.8105	**0.2191**	1.0852	1.5705	1.8006
	*MAPE*	5.0651	4.7792	**4.2920**	5.0193	5.1079
	*RMSE*	0.7390	0.6656	**0.6456**	0.7284	0.7450
	*RRMSE*	0.9915	**0.2058**	0.7777	0.8692	0.9670
	*t*_*sta*_	6.6253	5.9753	**5.7959**	6.5391	6.6882
	*R*^*2*^	0.9831	0.9845	**0.9871**	0.9831	0.9826
Zahedan	*MBE*	0.0835	-0.0528	-1.0076	0.0116	**0.0003**
	*MABE*	0.7254	0.7422	1.3964	0.7113	**0.6991**
	*MPE*	**-0.2309**	-1.5627	-9.1243	-0.8111	-0.9101
	*MAPE*	6.1416	6.0150	11.5803	6.0959	**6.0035**
	*RMSE*	0.8963	0.9303	2.1632	0.8723	**0.8630**
	*RRMSE*	7.1241	7.3943	17.1940	6.9360	**6.8592**
	*t*_*sta*_	0.3102	0.1886	1.7457	0.0443	**0.0012**
	*R*^*2*^	0.9741	**0.9820**	0.8730	0.9752	0.9762
Zanjan	*MBE*	0.1406	-0.2394	**0.0851**	0.1214	0.1138
	*MABE*	0.4692	0.3786	**0.3777**	0.4618	0.4737
	*MPE*	1.8047	-2.4273	**0.9689**	1.6030	1.5467
	*MAPE*	5.3609	**4.4691**	4.6437	5.2966	5.4114
	*RMSE*	0.5659	0.4488	**0.4285**	0.5584	0.5732
	*RRMSE*	5.8149	4.6119	**4.4038**	5.7386	5.8907
	*t*_*sta*_	0.8507	2.0920	0.6720	0.7385	**0.6719**
	*R*^*2*^	0.9862	**0.9936**	0.9921	0.9863	0.9854

**Table 7:** The best exergy models recognized for the eight selected locations of Iran.

Location	Functional form	Best model
Urmia	Cubic	$H_{Exergy}/H_{o}=-13.146{\left(n/N\right)}^{3}\;+26.337{\left(n/N\right)}^{2}-17.283\left(n/N\right)+4.024$
Boushehr	Power	$H_{Exergy}/H_{o}=0.380{\left(n/N\right)}^{0.380}$
Isfahan	Exponential	$H_{Exergy}/H_{o}=0.348{\text{e}}^{0.053\left(n/N\right)}$
Ilam	Exponential	$H_{Exergy}/H_{o}=0.334{\text{e}}^{0.210\left(n/N\right)}$
Kerman	Linear	$H_{Exergy}/H_{o}=0.323\left(n/N\right)+0.087$
Mashhad	Quadratic	$H_{Exergy}/H_{o}=-3.362{\left(n/N\right)}^{2}+5.275\left(n/N\right)-1.652$
Zahedan	Power	$H_{Exergy}/H_{o}=0.421{\left(n/N\right)}^{0.190}$
Zanjan	Cubic	$H_{Exergy}/H_{o}=-10.998{\left(n/N\right)}^{3}+22.973{\left(n/N\right)}^{2}-15.643\left(n/N\right)+3.791$

#### 3.3.1 Urmia

The results show that for Urmia, the cubic model has the least error in terms of six indicators of *MBE*, *MABE*, *MPE*, *MAPE*, *RMSE* and *RRMSE*. According to *R*^2^ all models have shown almost a similar performance, but cubic model has performed slightly better. The *t*_sta_ indicator for the power model is 0.2049, while for the cubic model it is 0.3657. However, in overall the cubic model is recognized as the best exergy model for Urmia.

#### 3.3.2 Boushehr

For Bushehr, the power model in terms of five indicators of *MPE*, *MBE*, *RMSE*, *RRMSE* and *t*_sta_, provides the least error and according to *R*^2^ is the best model. However, the *MABE* and *MAPE* of the exponential model are slightly better. The *MABE* and *MAPE* for the exponential model are 0.8939 MJ/m^2^day and 9.0018, respectively, but for the power model they are 0.8963 MJ/m^2^day and 9.0120, respectively. However, in overall the power model seems to be the best exergy model for Boushehr.

#### 3.3.3 Isfahan

For Isfahan, based on the four indicators of *MABE*, *MAPE*, *RMSE* and *RRMSE* the exponential model has the least error, also is the best model in terms of *R*^2^. Then is the quadratic model with the least error according to *MBE*, *MPE* and *t*_sta_. However, in overall the exponential model is recognized as the best exergy model for Isfahan.

#### 3.3.4 Ilam

For Ilam, based on four indicators of *MABE*, *MAPE*, *RMSE* and *RRMSE* the linear model and based on three indicators of *MBE*, *MPE* and *t*_sta_ the exponential model have performed better than other models. Also according to *R*^2^, all models show a similar performance. In order to choose the best model among the linear and the exponential models careful comparison was made. The *MABE*, *MAPE*, *RMSE* and *RRMSE* indicators for the linear model are 0.4273 MJ/m^2^day, 4.2693%, 0.5219 MJ/m^2^day and 4.3780%, respectively, and for the exponential model are 0.4309 MJ/m^2^day, 4.2811%, 0.5227 MJ/m^2^day and 4.3845%, respectively; thus the differences are not significant. However, *MBE*, *MPE* and *t*_sta_ for the linear model are 0.0217 MJ/m^2^day, 0.9631% and 0.1381, respectively, while for the exponential model they are 0.0084 MJ/m^2^day, 0.8484% and 0.0530, respectively. As noticed the differences are notable, so it can be concluded that for Ilam the exponential model is the best exergy model.

#### 3.3.5 Kerman

For Kerman, based on *RMSE* and *RRMSE*, *t*_sta_ and *R*^2^ all models have shown almost a similar performance, nevertheless based on *MBE*, *MABE*, *MPE* and *MAPE* the linear model has performed better. Thus, the linear model is recognized as the best exergy model for Kerman.

#### 3.3.6 Mashhad

For Mashhad, based on three indicators of *MBE*, *MPE* and *RRMSE* the quadratic model and based on *MABE*, *MAPE*, *RMSE*, *R*^2^ and *t*_sta_ the cubic model seem superior to other models. The *MBE*, *MPE* and *RRMSE* indicators for the quadratic model are 0.0412 MJ/m^2^day, 0.2191%, and 0.2058%, respectively, and for the cubic model they are 0.1474 MJ/m^2^day, 1.0852% and 0.7777%, respectively. The *MABE*, *MAPE*, *RMSE* and *t*_sta_ indicators for the cubic model are 0.4917 MJ/m^2^day, 4.2920%, 0.6456 MJ/m^2^day and 5.7959, respectively, and for the quadratic model they are 0.5434 MJ/m^2^day, 4.7792%, 0.6656 MJ/m^2^day and 5.9753, respectively. The difference between the *R*^2^ of the quadratic and that of the cubic models is only 0.003. Also the differences noticed for *MABE*, *MAPE*, *RMSE* and *t*_sta_ are not significant; thus in overall the quadratic model is the best exergy model for Mashhad.

#### 3.3.7 Zahedan

For Zahedan *MBE*, *MABE*, *MAPE*, *RMSE*, *RRMSE* and *t*_sta_ suggest that the power model is superior to other models. Although the quadratic model provides a slightly better *R*^2^ and the linear model provides a better *MPE*, in overall the power model is the best exergy model for Zahedan.

#### 3.3.8 Zanjan

For Zanjan, six indicators of *MBE*, *MABE*, *MPE*, *RMSE*, *RRMSE* and *t*_sta_ show that the cubic model has performed better than other calibrated models. Although, the *MAPE* and *R*^2^ of the quadratic model are slightly better than those of the cubic model, still the cubic model is the best exergy model for Zanjan.

## 4 Conclusion

The global solar radiation is the main driving force for all environmental processes on the earth as well as application of solar systems; therefore it is of great importance. More important is the quality of solar radiation, which determines the maximum possible work output that can be produced by solar radiation at a particular place, called solar radiation exergy. In this study, in order to improve the general information about solar exergy distribution around the globe and to study the status of solar exergy in Iran in particular, the solar and metrological data of eight stations of Iran were utilized. These stations, which are capitals of eight Iranian provinces, are Urmia, Boushehr, Isfahan, Ilam, Kerman, Mashhad, Zahedan and Zanjan. The monthly average daily solar radiation exergy on a horizontal surface for each station was obtained first. Then, for each station five models were calibrated and finally the best model was determined utilizing the statistical indicators.

The most important results of the present study are:

I. Long term measured data indicated that the relative sunshine duration for three stations of Isfahan, Kerman and Zahedan are higher than those of other stations. The maximum monthly average daily solar radiation is 21.22 MJ/m^2^day on July for Kerman and the minimum is 6.23 MJ/m^2^day on November for Zanjan.
II. The maximum monthly average daily solar radiation exergy is 18.51 MJ/m^2^day in Kerman on July and the minimum is 5.47 MJ/m^2^day in Zanjan on November.
III. The results show that the ratio of exergy to energy is almost independent of the month, climatic conditions and the geographical location of the nominated stations, such that this ratio can be considered 0.87 for the whole Iran.
IV. The best models for predicting the monthly average solar exergy in Urmia, Bushehr, Isfahan, Ilam, Kerman, Mashhad, Zahedan and Zanjan are cubic, power, exponential, exponential, linear, quadratic, power and cubic, respectively.
V. Similar to the Angstrom-Prescott model, which is to predict the total solar radiation, the best solar exergy models established in this study are dependent solely to the monthly average daily relative sunshine duration, but independent of any other solar or metrological parameter; hence are simple and easy to apply.
VI. The best models recognized for the nominated stations of this study may be used for other places with similar climatic classification and conditions around the globe, as those of the nominated stations.

## Declarations

### Author contribution statement

Hossein Khorasanizadeh: Conceived and designed the experiments; Performed the experiments; Analyzed and interpreted the data; Contributed reagents, materials, analysis tools or data; Wrote the paper.

Mojtaba Sepehrnia: Performed the experiments; Analyzed and interpreted the data; Wrote the paper.

### Funding statement

This work was supported by the 10.13039/501100005783University of Kashan (Grant No. 1397/1).

### Data availability statement

Data will be made available on request.

### Declaration of interests statement

The authors declare no conflict of interest.

### Additional information

No additional information is available for this paper.
